# Malignant Growths in the Mouth

**Published:** 1904-12

**Authors:** R. H. M. Dawbarn

**Affiliations:** New York


### MALIGNANT GROWTHS IN THE MOUTH.[1]

BY R. H. M. DAWBARN, M.D., NEW YORK.

Gentlemen,—The chief forms of malignancy directly of interest to stomatologists are, the bony tumors of the jaws, malignant epulis, cancer or sarcoma of the tonsils or pharynx, cancer of the tongue and of the lip.

In order to compress my paper within modest limits I shall only refer to such points in diagnosis as have a very practical bearing for you. As to tumors of the jaws, the case narrated by Dr. Howe demonstrates the ease with which a growth filling the antrum can be overlooked. I show you to-night the jaw and antrum tumor of this patient sent me by Dr. Howe about seven years ago upon a shrewd suspicion. It proved spindle-celled sarcoma, was excised, and remains cured. The use of a stout needle in this case, held in a firm holder, at once revealed decalcification of the bone. This you can verify for yourselves upon this specimen. The electric-light test also showed the antrum filled on that side. Another case, sent me last December by Dr. W. C. Dears, ex-president of the First District Dental Society, presented nothing but a very trifling swelling of the bony roof of the mouth; but the needle test showed, where there should have been almost stony hardness, a softened area, about as firm as articular cartilage and shading gradually into the surrounding dense bone. This area was considerably larger than a silver dollar and irregular in outline. I operated upon her at the Polyclinic Hospital in December, 1903, removing by drill and rongeur the softened area, also about one centimetre of dense bone beyond, for safety, and of course the same extent of periosteum and mucous membrane. I could then have passed both thumbs into the nose through the gap so left. Professor Jeffries, pathologist at this school, pronounced the pieces of softened bone sent him typical carcinomatous.

At first, after healing, this lady wore a temporary plate, for obvious reasons; but by continuous maintenance of unhealed edges, thus inviting growth by granulation, I have almost closed the gap. It is now not larger than the head of a pin. She is courteous

[1] Read before The New York Institute of Stomatology, June 3, 1904.

enough to be present to-night for your examination. So far as I can ascertain, early decalcification, permitting successful use of the needle test, is the rule rather than the exception; but it has not heretofore been recognized that such is the case. I have proved it in a considerable number of instances now; but one each of sarcoma and carcinoma of the upper jaw suffice to-night to remind you of it.

As to cancer of the tongue, unquestionably neglect of decaying teeth, sharp-edged and persistently irritating the tongue against their lingual surface, constitutes the chief predisposing cause. The cancer begins almost always at the edge of the tongue, continually chafed by such a tooth; and here, as in epulis, or, indeed, any other soft growth, it is wise for the dentist to inject a few drops of, say, one per cent. solution of cocaine, and then with curved scissors and forceps remove a small piece for the opinion of an expert in pathology. Then you will know what to expect, as to prognosis, after your surgeon has operated; and this report will also be his guide as to how extensive must be his excision.

Turning to treatment, we may put it rather concisely by saying that as yet there is no known means of a medical nature which is curative of malignancy; although arsenic pushed as far as is safe, by hypodermic, unquestionably slows and even checks the growth for a time of certain forms of sarcoma.

Prompt and wide excision remains our only safeguard, and by early diagnosis you have it in your power to save many lives through wise advice not to delay operation. But if the tumor be too extensive when first seen, or if it be so placed as to make excision anatomically hopeless, then our resources are either radium, X-rays, or Finsen light, naming them in order of speedy efficacy; or in just one instance, certain fusiform-celled sarcomata, the Coley antitoxin treatment; and finally my own plan of attempted starvation by depriving the tumor of its nourishment.

Regarding the use of radium and X-rays, doubtless Dr. Franklin will speak fully; but it is the opinion of surgeons that where excision is possible it is unwise to delay. There have been some really brilliant cures by these means; but, on the other hand, I have seen several cases go from bad to worse, and beyond hope of excision, during the delay of several weeks or longer with X-ray treatment. We cannot yet say why one case is helped and another is not. Apparently radium or X-rays are more hopeful in sarcoma than in carcinoma; but even in the latter I have noted a marvellous relief from the agonizing pain of cancer of the bodies of the vertebræ by placing a small radium tube of great power for an hour in contact with the back. Fully twenty-six hours of freedom from suffering followed; and this could not have been merely psychical, for the patients did not even know that anything unusual was to be tried.

Our wisest plan, in cases still operable, is to excise; and after the wound has healed, to begin X-ray or radium treatment several times a week for a month or more, hoping in this way to prevent recurrence. Of late I have been following this plan quite regularly; and so do most other surgeons, I think; and not alone about the mouth, but after removal of the uterus or breast for cancer, or, indeed, wherever it may chance to have been.

But if the tumor cannot be excised, and if the other two plans indicated have failed, what then? Are we at the end of our rope? In the region supplied by the external carotid we are not; and it means much to a patient to know this, and thereby to substitute for the certainty of death once more the uncertainty of life!

When first I began experimenting in this unknown field, just nine years ago, I feared that attempted starvation of malignancy would result also in starving the nose, tongue, or other normal parts. Fortunately, this is untrue; and we now know that normal flesh is able to survive with surprisingly little blood—much less than is necessary in order to permit so very vascular a thing as a cancer to continue growing. Also, it was demonstrated that no good would result from merely tying the external carotid on one or even on both sides. Nothing short of complete excision of both these arteries was effective—doing it for safety at two different periods of time.

At present the writer has excised the external carotids in nearly sixty instances. The operation is quite surprisingly safe. If the patient is not badly cachectic, and if the surgeon is not so unwise as to attempt removal of the growth at the same sitting, there ought to be not over five per cent. of mortality. And simple ligation of both external carotids should have hardly any mortality at all.

What are the results?

In carcinoma, which spreads, as we all remember, not through the blood-vessels, but through its lymphatic channels, the results have been disappointing. Even here, however, we can expect with some confidence a considerable degree of shrinkage, but lasting only a few weeks or months,—up to a year in one case, who otherwise would certainly have died within a month. But in sarcoma there is reason for a more cheerful view; and, remembering that this malignant tumor spreads by its blood-vessels rather than its lymphatics, one could guess, *a priori*, that sarcoma would be better controlled by the starvation of blood than would carcinoma.

In Butlin, "On Malignant Disease," round-celled subperiosteal sarcoma of the jaws is considered hopeless, whatever we do; and yet in my Gross prize book are recorded two cases of this nature which have remained shrunken for much longer than the Volkmann period of three years, after which we may with some degree of certainty claim a permament cure, and which still remain quiescent. The tumor did not, of course, disappear, but it became much smaller, harder, and inactive. In several other instances of sarcoma there has been only a temporary checking; but this is generally longer than we have learned to expect in carcinoma. We must recollect that if a growth is so situated that it can get blood from the *internal* carotid or its branches,—about the orbit, for example,—the prognosis is necessarily bad. Of course, we cannot tie off the internal carotid too, for this would be useless unless done on both sides, and that would kill the patient promptly from anæmia of the brain. (Roughly, the internal carotid may be said to supply brain and eye; the external carotid all parts of the upper neck, and of the head except as just stated.)

Here in New York about a dozen members of the New York Surgical Society have tried my plan, and sent me their results, all of which have been published. In every instance of recovery from the operation itself cancer—*i.e.*, carcinoma—has been the form of malignancy; and, as it happens, in no case sarcoma as yet. This is unfortunate; and in fairness to this plan it is evident that it should be given a trial in sarcoma.

I trust it is plain to this society that this operation is not recommended by me for trial except as a last resource—after other things fail; and since a coffin is the only alternative, surely it is worthy of trial. In many instances patients are eager if only for a few weeks' longer life; perhaps for business reasons, to straighten out their affairs; and this at the very least we can give them. Again, in a case inoperable through size, and yet not extremely so, it is quite likely that we may be able, because of a few weeks' shrinkage, so to reduce the mass that it can safely be subjected to thorough work by the knife. I have within the past fortnight received from Professor J. Chalmers Da Costa, of Philadelphia, the account, sent me for publication, of a case of this very sort, refused as impossible of excision by two other prominent surgeons, but made easily operable by the starvation plan; so that after a few weeks' shrinkage Dr. Da Costa safely cut out the now shrunken tumor.

These points, then, we can surely claim for this method of the *dernier ressort*, and, in addition, in sarcoma, in some cases a real and permanent cure, in the sense that the shrunken mass has remained shrunken for years, and, so far as I know, permanently. The case sent the writer by Dr. Howe is such an instance, definitely cured; only there we could excise the tumor as well as the carotids.

In two cases in my book, of especial interest to you, as involving the lower jaw, one detail of the operation I must particularly mention and recommend. This is, upon completing the excision of the external carotid, to do a short, blunt dissection in the front of the wound high up, thus exposing the inner surface of the ramus and the inferior dental nerve and artery as they are about to enter the canal. The artery and also its mylohyoid or main branch, at this point easily seen, are tied. Thus we deprive the bone of a free anastomosis, for the inferior dental, coming from the internal maxillary, anastomoses freely through orbital branches with the ophthalmic from the internal or deep carotid. In cases of lower-jaw malignancy starvation, where this ligation is omitted, we can well see how the excision of the external carotid alone would prove almost valueless. This step does not add more than a few minutes to the length of the operation. Finally, in each of these cases an inch or more of the inferior dental nerve was excised; for, in case the growth in the mandible should start up again at some future time, at least we have made sure by this means that there will be little or no suffering. Of course, it is essential that this be done upon both sides of the neck, just as with the work upon the arteries; otherwise little is gained.

In conclusion we may claim the following advantages from the starvation plan in the external carotid area, in cases wisely selected.

1. In some instances of sarcoma it saves life by permanent shrinkage of a tumor too large to excise, or so placed that this is impossible.

2. In all instances surviving the carotid excisions there is a prolongation of life not otherwise possible of accomplishment; even in carcinoma this is true. This may in unfavorable cases be only a few months' gain, but patients are grateful even for this time in which the better to arrange their affairs.

3. Every operator has agreed that one striking effect is the lessening of pain, which is a prompt result of the anæmia produced.

4. In some instances the shrinkage permits the removal of a tumor not possible of excision because of its size and vascularity at first.

